# Aspirin Use Does Not Significantly Reduce the Risk of Liver Fibrosis in Patients With Metabolic Dysfunction‐Associated Steatotic Liver Disease

**DOI:** 10.1002/fsn3.71384

**Published:** 2025-12-26

**Authors:** Linlin Yin, Bin Zhang

**Affiliations:** ^1^ Department of Digestive Diseases, the Second Hospital of Nanjing Affiliated to Nanjing University of Chinese Medicine Nanjing Jiangsu China

**Keywords:** aspirin, liver fibrosis, MASLD, Mendelian randomization, NHANES database

## Abstract

The effect of aspirin on liver fibrosis is still controversial. To further explore the effect of aspirin on liver fibrosis in metabolic dysfunction‐associated steatotic liver disease (MASLD), we conducted this study. We applied NHANES database data to evaluate the degree of liver fibrosis through fibroscan and explored the impact of aspirin on liver fibrosis in patients with MASLD from a cross‐sectional study based on the American population. Considering the shortcomings of cross‐sectional studies, we also applied Mendelian randomization (MR) analysis, a novel research method, to evaluate the potential causal relationship of aspirin on liver fibrosis and cirrhosis from a genetic perspective. In a cross‐sectional study of the NHANES database, we did not find that aspirin significantly improved the prognosis of liver fibrosis in MASLD patients after adjusting for possible confounding biases by multivariable logistic regression analysis. Similarly, in univariate and multivariate MR analyses, we did not observe a potential causal relationship between aspirin and liver fibrosis or cirrhosis. Our study did not find that aspirin significantly reduced the risk of liver fibrosis in MASLD patients based on cross‐sectional studies of the American population and genetic associations by MR analysis.

## Graphical abstract

Our study did not find that aspirin significantly reduces the risk of liver fibrosis in patients with MASLD. Therefore, the long‐term use of aspirin solely for the prevention of liver fibrosis progression cannot be recommended in patients without high cardiovascular or cerebrovascular risk.

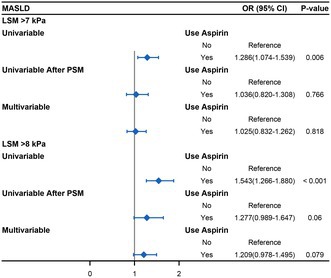

## 1 Introduction

Nonalcoholic fatty liver disease (NAFLD) is the most common chronic liver disease worldwide (Chalasani et al. 2018). To more accurately reflect the metabolic etiology of the disease and eliminate the potential stigmatization associated with the term “non‐alcoholic”, the term metabolic dysfunction‐associated steatotic liver disease (MASLD) was introduced in June 2023 to gradually replace NAFLD (Rinella et al. 2024). Currently, the global prevalence of MASLD is about 25%, among which the estimated prevalence in Asia and the United States is 27.4% and 30%, respectively (Younossi et al. 2016, 2018). Liver fibrosis due to metabolic dysfunction‐associated steatohepatitis (MASH) is very common, and its incidence has shown a clear upward trend in the United States in recent years (Kim et al. 2019). As of 2015, about 3.3 million people in the United States suffer from advanced fibrosis (Estes et al. 2018). It is estimated that the number of MASLD‐related deaths will increase by 178% between 2015 and 2030 (Estes et al. 2018). Similarly, in recent years, the rate of liver transplantation caused by MASH has increased 7‐fold in the United States and 7.7‐fold in Europe, which greatly increases the corresponding medical burden (Younossi et al. 2019; Haldar et al. 2019).

However, there is no effective drug treatment for MASLD and MASLD‐related liver fibrosis. Previous studies demonstrated that long‐term oral aspirin could reduce the progression of liver fibrosis in patients with chronic liver disease. Related animal studies suggest that aspirin can exert its anti‐fibrosis effect by inhibiting the TGF‐β‐1 pathway, proinflammatory cytokines IL‐1β and COX‐2 (Wu et al. 2023). A prospective study by Simon et al. showed that people who took daily aspirin had a significantly lower risk of advanced fibrosis among patients with MASLD (Simon et al. 2019). Similarly, a study based on the American population showed that aspirin use was associated with a significant decrease in liver fibrosis index in American adults with chronic liver disease (Jiang et al. 2016). However, in the AMIS (Aspirin‐Myocardial Infarction Study) trial, taking large doses of aspirin daily did not have a significant benefit for liver fibrosis (Tiwari‐Heckler et al. 2019).

The gold standard for the diagnosis of liver fibrosis is liver biopsy, but liver biopsy is an invasive and expensive examination, and there is a risk of serious complications such as bleeding and infection; so in clinical application, the evaluation of liver fibrosis is mainly through non‐invasive examination. The main non‐invasive examinations to assess liver fibrosis include transient elastography (FibroScan), platelets ratio index (APRI), fibrosis‐4 index (FIB‐4), BARD score, and NAFLD fibrosis score (NFS) (Xiao et al. 2017). Among them, transient elastography with controlled attenuation parameter (CAP) has higher accuracy in evaluating the degree of liver fibrosis in MASLD patients. A meta‐analysis study showed that FibroScan has high sensitivity and specificity in identifying different stages of liver fibrosis in MASLD (Fibrosis stage ≥ F2 [sensitivity: 62%–90%, specificity: 74%–100%], Fibrosis stage ≥ F3 [sensitivity: 84%–100%, specificity: 83%–97%], Fibrosis stage F4 [sensitivity: 90%–100%, specificity: 75.9%–98.4%]) (Mikolasevic et al. 2016).

Considering previous studies, the effect of aspirin on liver fibrosis is controversial. To further explore the potential antifibrotic benefits of aspirin in patients with MASLD, we first used the relevant data from the National Health and Nutrition Examination Survey (NHANES) to evaluate the effect of aspirin on liver fibrosis in patients with MASLD by FibroScan. Besides, considering that these traditional observational studies contain inherent defects such as confounding bias and reverse causality. Therefore, we used a novel causal inference method (MR) to explore the potential causal relationship between aspirin and liver fibrosis. Mendelian randomization, based on the random assignment of genetic variants during sperm and ovum formation, is similar to randomized controlled trial studies with less potential for confounding. MR needs to meet the following critical assumptions: (1) instrumental variables (IVs) are closely associated with exposure (aspirin); (2) IVs can only affect the outcome through exposure; and (3) instrumental variables are not related to outcome variables (liver fibrosis) and not be affected by confounding factors (Emdin et al. 2017).

## 2 Methods

### 2.1 Study Subjects

#### 2.1.1 NHANES Database Data

**FIGURE 1:**
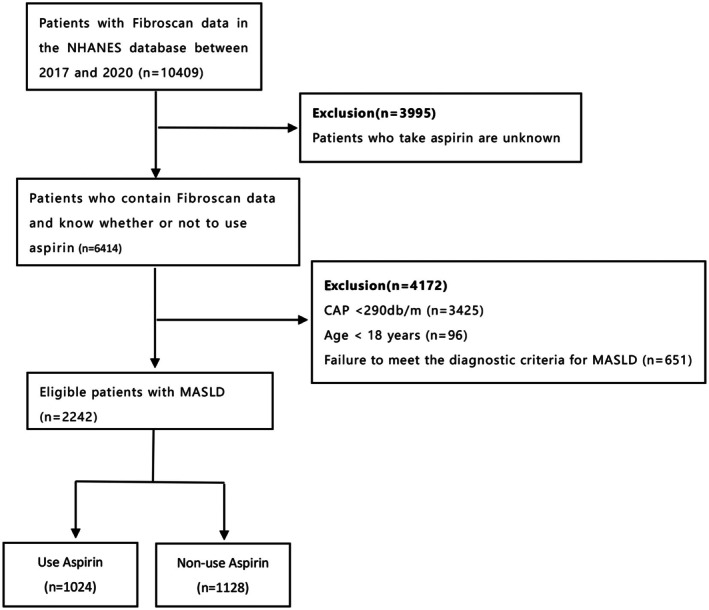
Flow diagram of eligible patients diagnosed with MASLD.

We collected Fibroscan data of 10,409 patients in the NHANES database from 2017 to 2020. NHANES is an ongoing program implemented by the National Center for Health Statistics (NCHS) of the Centers for Disease Control (CDC) to assess the health and nutritional status of the non‐institutionalized civilian population in the United States. We formulated the following exclusion criteria: (1) Failure to meet the diagnostic criteria for MASLD. (2) Age < 18 years. (3) Median value of controlled attenuation parameters (CAP) < 290db/m. (4) Patients who take aspirin are unknown. Finally, a total of 2242 MASLD patients from the NHANES database were included in this study (Figure 1).

#### 2.1.2 MR Analysis Data

The SNPs of aspirin came from the UK Biobank database involving 462,933 European population (61,702 cases vs. 401,231 controls). We initially selected SNPs that met the following conditions for MR analysis: significant association (*p* < 5 × 10–8); no obvious linkage disequilibrium (LD: *R*2 < 0.001). After preliminary screening, 14 SNPs were identified for aspirin. *R*2 represents the percentage of variation explained by SNPs, and the genetic instrumental variables of aspirin explained 0.15% phenotypic variation. Besides, we calculated *F* statistics to assess the strength of SNPs as instrumental variables and excluded IVs with *F* < 10 to reduce potential weak IV bias (*F* = *R*2 (*N* − 2)/(1 − *R*2); *N* represents the sample size, and *R*2 refers to the variance of exposure explained by IVs).

GWAS summary statistics for aspirin with fibrosis and cirrhosis of the liver were identified from the FinnGen study. Finally, our study comprised fibrosis and cirrhosis of the liver (1362 cases vs. 301,014 controls) from the FinnGen study.

### 2.2 Definition of MASLD and Significant Fibrosis

MASLD was defined as the presence of hepatic steatosis (CAP ≥ 290 db/m) plus at least 1 of 5 criteria: (1) BMI ≥ 25 kg/m^2^ (≥ 23 kg/m^2^ in Asian), (2) prediabetes or diabetes, (3) hypertension, (4) high triglycerides, and (5) low HDL cholesterol (Thrift et al. 2023) For significant liver fibrosis, we chose the cutoff value of LSM (liver stiffness measurement) > 7 kPa. In the sensitivity analysis, according to the recent guidance of EASL, we used a higher cutoff value (LSM > 8 kPa) for significant liver fibrosis (European Association for the Study of the Liver 2021).

### 2.3 Statistical Analysis

We first compared the clinical characteristics of patients in the non‐use aspirin group and the use aspirin group by chi‐square test. Second, we explored the association between aspirin and significant liver fibrosis (LSM > 7 kPa) in MASLD patients by univariate logistic regression analysis and multivariate logistic regression after adjusting for potential clinical confounders. In addition, propensity score matching (PSM) was used to reduce selection bias and confounding variables between the aspirin use group and the non‐aspirin use group. A 1:1 PSM was implemented according to age, gender, race, marital status, smoking history, comorbid diseases (hypertension, diabetes, hypertriglyceridemia, high‐low‐density cholesterol, low‐high‐density cholesterol, coronary heart disease, stroke), and BMI. The caliper value was set to 0.02. The difference in the occurrence of significant liver fibrosis between the two groups after matching was compared again. For further sensitivity analysis, we also analyzed the association between aspirin and significant fibrosis (LSM > 8 kPa) in MASLD patients. Finally, we also analyzed the potential causal relationship between aspirin and liver fibrosis and cirrhosis by means of univariate MR analysis and multivariate MR analysis.

We apply the “TwoSampleMR” package and the “MRPRESSO” package for MR analysis. The MR results are mainly analyzed by the random effects inverse variance weighted (IVW) method. In addition, weighted median and MR‐Egger methods were applied to further assess the robustness of our results. We also used Cochran's *Q* test to assess heterogeneity and assessed horizontal pleiotropy by MR‐Egger intercept method and MR‐PRESSO method. Meanwhile, we also applied leave‐one‐out SNP analysis to assess whether the observed associations were influenced by a single SNP.

We use the following software for the above analysis: SPSS25.0 for the Chi‐squared test, logistics regression model. PSM and MR analysis were performed in R software 4.1.0. All statistical tests were double‐tailed distribution; *p* < 0.05 is considered to be significant.

## 3 Results

### 3.1 Clinical Characteristics

After screening, the NHANES database included a total of 2242 MASLD patients. Among them, there were 1024 (45.7%) patients in the use‐aspirin group and 1218 (54.3%) patients in the non‐use‐aspirin group. The Table 1 provides the baseline characteristics of the two groups of patients. We found that there were significant differences between the non‐use‐aspirin group and the use‐aspirin group in age, sex, race, marital status, income, hypertension, diabetes, CAD, stroke, smoking history, and LDL cholesterol. Patients taking aspirin were more likely to be age ≥ 65 (47.8% vs. 20.3%, *p* < 0.001), man (59.4% vs. 50.6%, *p* < 0.001), Non‐Hispanic White (44.1% vs. 32.9%, *p* < 0.001), divorced (28.2% vs. 24.8%, *p* = 0.028) and INDFMMPI > 1.85 (51.6% vs. 45.3%, *p* = 0.001) than the non‐use‐aspirin group. At the same time, we found that the proportion of hypertension (71.9% vs. 43.8%, *p* < 0.001), diabetes (42.7% vs. 18.1%, *p* < 0.001), CAD (13.1% vs. 1.5%, *p* < 0.001), stroke (9.5% vs. 2.9%, *p* < 0.001), former smoking (36.1% vs. 26.7%, *p* < 0.001), and non‐high LDL cholesterol (70.3% vs. 61.3%, *p* < 0.001) in the use‐aspirin group was higher than that in the non‐use‐aspirin group. Besides, compared with the non‐use‐aspirin group, significant liver fibrosis was more common in the use‐aspirin group (LSM > 7 kPa: 33.7% vs. 28.3%, *p* = 0.006; LSM > 8 kPa: 27.1% vs. 19.5%, *p* < 0.001).

**TABLE 1:** Baseline characteristics of patients with MASLD before and after PSM.

Variables	Before PSM		*p*	After PSM		*p*
	Non‐use aspirin	Use aspirin		Non‐use aspirin	Use aspirin	
	*n* = 1218	*n* = 1024		*n* = 653	*n* = 653	
Age group, years, %						
< 65	971 (79.7%)	535 (52.2%)	< 0.001	440 (67.4%)	426 (65.2%)	0.412
≥ 65	247 (20.3%)	489 (47.8%)		213 (32.6%)	227 (34.8%)	
Sex, %						
Male	616 (50.6%)	608 (59.4%)	< 0.001	346 (53.0%)	353 (54.1%)	0.698
Female	602 (49.4%)	416 (40.6%)		307 (47.0%)	300 (45.9%)	
Marital status, %						
Married	784 (64.4%)	653 (63.8%)	0.028	409 (62.6%)	412 (63.1%)	0.873
Divorced	302 (24.8%)	289 (28.2%)		181 (27.7%)	174 (26.6%)	
Unmarried	132 (10.8%)	82 (8.0%)		63 (9.6%)	67 (10.3%)	
Race, %						
Mexican American	204 (16.7%)	112 (10.9%)	< 0.001	94 (14.4%)	79 (12.1%)	0.159
Other Hispanic	134 (11.0%)	110 (10.7%)		81 (12.4%)	78 (11.9%)	
Non‐Hispanic White	401 (32.9%)	452 (44.1%)		228 (34.9%)	266 (40.7%)	
Non‐Hispanic Black	279 (22.9%)	229 (22.4%)		154 (23.6%)	149 (22.8%)	
Non‐Hispanic Asian	152 (12.5%)	78 (7.6%)		76 (11.6%)	56 (8.6%)	
Other Race	48 (3.9%)	43 (4.2%)		20 (3.1%)	25 (3.8%)	
Education, %						
Less than College graduate	538 (44.2%)	460 (44.9%)	0.721	284 (43.5%)	288 (44.1%)	0.823
College graduate or above	680 (55.8%)	564 (55.1%)		369 (56.5%)	365 (55.9%)	
Income, %						
INDFMMPI ≤ 1.30	349 (28.7%)	241 (23.5%)	0.001	180 (27.6%)	158 (24.2%)	0.051
1.30 < INDFMMPI ≤ 1.85	168 (13.8%)	161 (15.7%)		86 (13.2%)	105 (16.1%)	
INDFMMPI > 1.85	552 (45.3%)	528 (51.6%)		300 (45.9%)	327 (50.1%)	
Unknown	149 (12.2%)	94 (9.2%)		87 (13.3%)	63 (9.6%)	
Hypertension, %						
Yes	533 (43.8%)	736 (71.9%)	< 0.001	435 (66.6%)	398 (60.9%)	0.219
No	685 (56.2%)	288 (28.1%)		218 (33.4%)	255 (39.1%)	
Diabetes, %						
Yes	220 (18.1%)	450 (42.7%)	< 0.001	195 (29.9%)	210 (32.2%)	0.061
No	943 (77.4%)	530 (51.8%)		439 (67.2%)	402 (61.6%)	
Unknown	55 (4.5%)	44 (4.3%)		19 (2.9%)	41 (6.3%)	
CAD, %						
Yes	18 (1.5%)	134 (13.1%)	< 0.001	17 (2.6%)	26 (4.0%)	0.163
No	1200 (98.5%)	890 (86.9%)		636 (97.4%)	627 (96.0%)	
Stroke, %						
Yes	35 (2.9%)	97 (9.5%)	< 0.001	32 (4.9%)	41 (6.3%)	0.278
No	1183 (97.1%)	927 (90.5%)		621 (95.1%)	612 (93.7%)	
Smoking history, %						
Never	696 (57.1%)	515 (50.3%)	< 0.001	369 (56.5%)	336 (51.5%)	0.184
Former	325 (26.7%)	370 (36.1%)		197 (30.2%)	222 (34.0%)	
Active	197 (16.2%)	139 (13.6%)		87 (13.3%)	95 (14.5%)	
High LDL cholesterol, %						
Yes	399 (32.8%)	252 (24.6%)	< 0.001	197 (30.2%)	190 (29.1%)	0.514
No	747 (61.3%)	720 (70.3%)		418 (64.0%)	433 (66.3%)	
Unknown	72 (5.9%)	52 (5.1%)		38 (5.8%)	30 (4.6%)	
Low HDL cholesterol, %						
Yes	464 (38.1%)	368 (35.9%)	0.345	238 (36.4%)	233 (35.7%)	0.674
No	689 (56.6%)	609 (59.5%)		381 (58.3%)	392 (60.0%)	
Unknown	65 (5.3%)	47 (4.6%)		34 (5.2%)	28 (4.3%)	
Hypertriglyceridemia, %						
Yes	519 (42.6%)	478 (46.7%)	0.140	282 (43.2%)	292 (44.7%)	0.571
No	627 (51.5%)	494 (48.2%)		333 (51.0%)	331 (50.7%)	
Unknown	72 (5.9%)	52 (5.1%)		38 (5.8%)	30 (4.6%)	
BMI, %						
< 25 cm	49 (4.0%)	53 (5.2%)	0.192	29 (4.4%)	25 (3.8%)	0.578
≥ 25 cm	1169 (96.0%)	971 (94.8%)		624 (95.6%)	628 (96.2%)	
LSM > 7 kPa, %						
Yes	345 (28.3%)	345 (33.7%)	0.006	204 (31.2%)	209 (32.0%)	0.766
No	873 (71.7%)	679 (66.3%)		449 (68.8%)	444 (68.0%)	
LSM > 8 kPa, %						
Yes	237 (19.5%)	278 (27.1%)	< 0.001	142 (21.7%)	171 (26.2%)	0.060
No	981 (80.5%)	746 (72.9%)		511 (78.3%)	482 (73.8%)	

### 3.2 Univariate and Multivariate Logistic Regression Analysis

**FIGURE 2:**
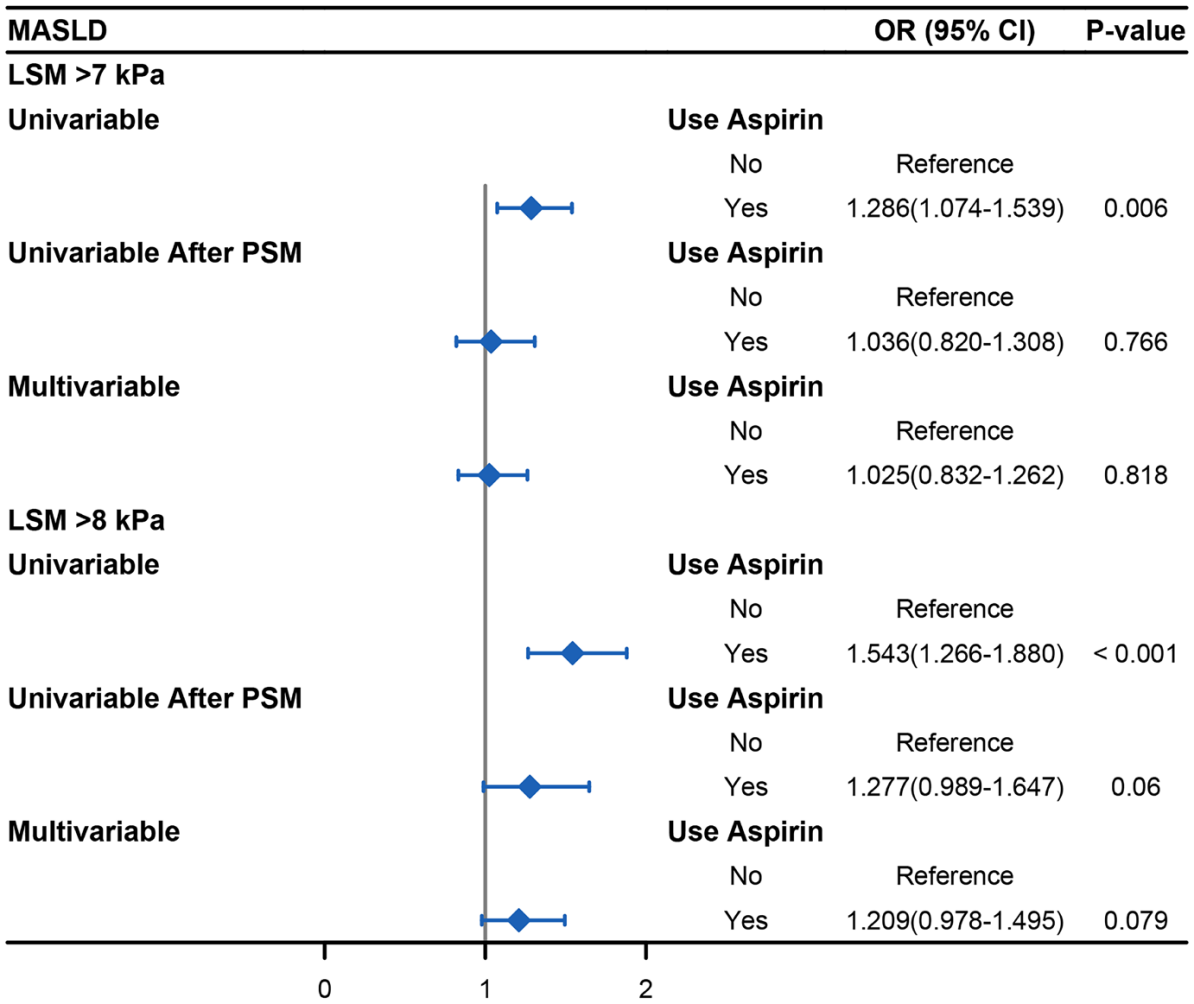
Forest plot of the effect of aspirin on the risk of liver fibrosis in patients with MASLD.

Univariate logistic regression analysis showed that long‐term oral aspirin increased the risk of liver fibrosis in MASLD patients (LSM > 7 kPa, OR: 1.286, 95% CI: 1.074–1.539, *p* = 0.006) (Figure 2). However, after adjustment for other variables (age, gender, marital status, race, income, education, smoking history, hypertension, diabetes, hypertriglyceridemia, high LDL cholesterol, low HDL cholesterol, CAD, stroke, and BMI) by multivariate logistic regression analysis, we found no significant association between aspirin and liver fibrosis (LSM > 7 kPa, OR: 1.025, 95% CI: 0.832–1.262, *p* = 0.818) (Table 2, Figure 2).

**TABLE 2:** Multivariate logistic regression analysis in patients with MASLD.

Characteristics	LSM > 7 kPa	*p*	LSM > 8 kPa	*p*
	Adjusted OR (95% CI)		Adjusted OR (95% CI)	
Age group, years				
< 65	Reference		Reference	
≥ 65	0.823 (0.669–1.012)	0.064	0.822 (0.647–1.044)	0.108
Sex				
Male	Reference		Reference	
Female	0.824 (0.679–0.999)	0.049	0.766 (0.62–0.948)	0.014
Marital status				
Married	Reference		Reference	
Divorced	1.112 (0.886–1.396)	0.358	1.124 (0.876–1.441)	0.359
Unmarried	1.221 (0.88–1.695)	0.232	1.135 (0.79–1.63)	0.493
Race				
Mexican American	Reference		Reference	
Other Hispanic	1.115 (0.772–1.611)	0.561	1.063 (0.707–1.599)	0.768
Non‐Hispanic White	0.909 (0.67–1.232)	0.537	1.001 (0.715–1.402)	0.995
Non‐Hispanic Black	1.045 (0.751–1.454)	0.792	1.108 (0.769–1.595)	0.582
Non‐Hispanic Asian	0.811 (0.537–1.226)	0.321	0.977 (0.623–1.534)	0.921
Other Race	0.878 (0.518–1.489)	0.629	1.083 (0.615–1.908)	0.781
Education				
Less than College graduate	Reference		Reference	
College graduate or above	1.124 (0.918–1.376)	0.258	1.016 (0.815–1.267)	0.887
Income				
INDFMMPI ≤ 1.30	Reference		Reference	
1.30 < INDFMMPI ≤ 1.85	0.946 (0.698–1.283)	0.722	0.956 (0.682–1.34)	0.795
INDFMMPI > 1.85	0.968 (0.76–1.233)	0.791	1.064 (0.816–1.388)	0.647
Hypertension				
Yes	Reference		Reference	
No	0.757 (0.623–0.919)	0.005	0.88 (0.703–1.101)	0.264
Diabetes				
Yes	Reference		Reference	
No	0.482 (0.393–0.591)	< 0.001	0.455 (0.364–0.567)	< 0.001
CAD				
Yes	Reference		Reference	
No	1.24 (0.842–1.824)	0.276	1.297 (0.859–1.96)	0.216
Stroke				
Yes	Reference		Reference	
No	0.872 (0.589–1.293)	0.496	0.808 (0.533–1.224)	0.314
Smoking history				
Never	Reference		Reference	
Former	1.03 (0.833–1.273)	0.785	0.938 (0.746–1.179)	0.582
Active	0.881 (0.669–1.16)	0.367	0.782 (0.574–1.064)	0.117
High LDL cholesterol				
No	Reference		Reference	
Yes	0.772 (0.615–0.968)	0.025	0.739 (0.581–0.94)	0.014
Low HDL cholesterol				
No	Reference		Reference	
Yes	1.436 (1.179–1.749)	< 0.001	1.381 (1.114–1.713)	0.003
Hypertriglyceridemia				
No	Reference		Reference	
Yes	0.984 (0.795–1.217)	0.879	0.997 (0.79–1.258)	0.979
BMI				
< 25 cm	Reference		Reference	
≥ 25 cm	2.539 (1.445–4.46)	0.001	1.847 (1.058–3.223)	0.031
Use aspirin				
No	Reference		Reference	
Yes	1.025 (0.832–1.262)	0.818	1.209 (0.978–1.495)	0.079

### 3.3 Propensity Score‐Matched

One‐to‐one matching between the aspirin group and the non‐aspirin group was performed with a caliper of 0.02. The propensity model included the following variables: age, gender, marital status, race, income, education, smoking history, hypertension, diabetes, hypertriglyceridemia, high LDL cholesterol, low HDL cholesterol, CAD, stroke, and BMI. After PSM, 653 matched pairs with similar baseline characteristics were included in our study (Table 1). The histogram that matches the score showed that the two groups fit well (Figure S1). Following the propensity‐score analyses, univariate logistic regression analysis found that the risk of liver fibrosis was comparable between the aspirin group and the non‐aspirin group (LSM > 7 kPa, OR: 1.036, 95% CI: 0.820–1.308, *p* = 0.766) (Figure 2).

### 3.4 Sensitivity Analysis

For further sensitivity analysis, we analyzed the association between aspirin and significant fibrosis (LSM > 8 kPa) in MASLD patients. The results of sensitivity analysis showed that aspirin was proportional to the risk of liver fibrosis by univariate logistic regression analysis in MASLD patients (LSM > 8 kPa, OR: 1.543, 95% CI: 1.266–1.880, *p* < 0.001) but after adjusting for confounding factors in multivariate logistic regression analysis, we found that there was no significant correlation between aspirin use and liver fibrosis (LSM > 8 kPa, OR: 1.209, 95% CI: 0.978–1.495, *p* = 0.079) (Table 2, Figure 2). Similarly, after matching the two groups of baseline data by PSM, the logistic regression results still showed that long‐term oral aspirin did not significantly reduce the degree of liver fibrosis in MASLD patients (LSM > 8 kPa, OR: 1.277, 95% CI: 0.989–1.647, *p* = 0.060) (Figure 2).

### 3.5 Results of MR Analysis

Two SNPs (rs1831733 and rs8126001) were excluded from VIs for aspirin, as they are not available in fibrosis and cirrhosis of the liver. Finally, 12 SNPs for aspirin were included in the MR analysis. Details on the instrumental variables for aspirin (exposure) can be found in Table S1, and their associations with fibrosis and cirrhosis of the liver (outcome) from the FinnGen study are given in Table S2.

The MR results using IVW indicated that aspirin could not significantly decrease the risk of liver fibrosis (OR: 0.259, 95% CI: 8.39^−5^–8.01^2^, *p* = 0.742). Similarly, in subsequent MR‐Egger (OR: 0.001, 95% CI: 4.02^−12^–3.14^5^, *p* = 0.511) and Weight Median estimator methods (OR: 0.204, 95% CI: 1.44^−5^–2.87^3^, *p* = 0.744), we found no significant evidence for the effect of gene‐predicted aspirin on fibrosis and cirrhosis of the liver. We used Cochran's *Q* test to identify heterogeneity across individual SNPs and no significant heterogeneity was found for the relationship between fibrosis and cirrhosis of the liver with all gene‐predicted aspirin (MR Egger: *Q* = 13.79, *p* = 0.13; IVW: *Q* = 14.35, *p* = 0.15). In the evaluation of horizontal pleiotropy, neither MR‐Egger intercept (egger intercept = 0.04, *p* = 0.56) nor MR‐PRESO (*p* = 0.861) found significant horizontal pleiotropy. Besides, in the leave‐one‐out analysis, we found no significant changes in risk estimates for aspirin and risk of fibrosis and cirrhosis of the liver after excluding 1 SNP at each time (Figure S2). After we adjusted for these possible confounding factors (Alcoholic liver disease, viral liver disease, diabetes), the multivariate MR analysis result showed that there was no obvious causal relationship between aspirin and chronic liver fibrosis (IVW OR: 0.796, 95% CI: 0.012–53.594, *p* = 0.915).

## 4 Discussion

One of the main goals of treatment for patients with MASLD is to prevent and delay the occurrence and progression of liver fibrosis. For the occurrence of liver fibrosis in chronic liver disease, it is difficult to have drugs to reverse the occurrence of fibrosis. Accumulating evidence showed that aspirin had a potential anti‐hepatic fibrosis effect.

However, in our study, oral aspirin was not found to significantly reduce the progression of liver fibrosis in patients with MASLD. Meanwhile, we found that more patients in the oral aspirin group had hypertension, diabetes, coronary heart disease, stroke, and former smoking. Previous studies have shown that hypertension is a high‐risk factor for the progression of liver fibrosis in MASLD patients (Man et al. 2023; Suárez et al. 2023). Saori et al. demonstrate that diabetes‐induced hypoxia and oxidative stress damage central liver sinusoidal endothelial cells in zone 3 hepatocytes, which may mediate inflammation and stellate cell activation, leading to the progression of liver fibrosis (Sako et al. 2023). The related research also suggests that smoking can damage the glutathione antioxidant pathway, leading to free radical lipid peroxidation, which further leads to inflammation of liver cells, and activation of hepatic stellate cells and mesangial cells, thereby increasing fibrosis mediators to further aggravate the Progression of liver fibrosis (Premkumar and Anand 2021). Considering the presence of these confounding factors that increase the progression of liver fibrosis in the aspirin group, this may explain the significantly increased risk of liver fibrosis in the aspirin group in our univariate logistic regression analysis. After adjusting the confounding factors by multivariate logistic regression analysis, our results showed that oral aspirin did not reduce the risk of liver fibrosis and hypertension, diabetes, Low HDL cholesterol, BMI ≥ 25 significantly increased the risk of liver fibrosis, which further confirmed this point. After adjusting the baseline data by PSM, it provided further evidence that oral aspirin did not reduce the risk of liver fibrosis progression in MASLD patients. Our subsequent Mendelian randomization analysis also failed to find a significant causal relationship between aspirin and chronic liver fibrosis.

Our study has the following advantages. Firstly, our study explored the relationship between aspirin and liver fibrosis from two perspectives (population‐based study and Mendelian randomization study), and the consistency of the two results strengthens the persuasive conclusion of our study. Secondly, we included a large sample of patients, including two groups (the United States and Europe), which made our results more universal. In addition, we use multivariate logistic regression analysis, PSM, and multivariate MR analysis to reduce confounding bias to make our study more reliable. However, our study still has the following shortcomings. Firstly, the NHANES database is retrospective and further prospective studies are needed to explore the effects of aspirin on liver fibrosis. Secondly, the aspirin consumption data comes from the questionnaire survey in NHANES, and we do not know the specific dosage and time of aspirin taken by the patients. The preventive effect of aspirin on liver fibrosis may be time‐ and dose‐dependent, and short‐term and low‐dose oral aspirin may not be able to improve and delay the progress of liver fibrosis. Thirdly, our study lacked other non‐invasive tools for the assessment of liver fibrosis, such as the Enhanced Liver Fibrosis (ELF) score. The ELF score has now been recommended as a screening tool for liver fibrosis in patients with MASLD by the Asia–Pacific clinical practice guidelines, as well as by the joint guidelines issued by the European Association for the Study of the Liver (EASL), the European Association for the Study of Diabetes (EASD), and the European Association for the Study of Obesity (EASO) (EASL, EASD, and EASO 2024; Sterling et al. 2025; Eslam et al. 2025). Moreover, ELF is currently the only FDA‐approved non‐invasive prognostic biomarker for predicting hepatic decompensation (Castera et al. 2025). Owing to the unavailability of key components required for ELF calculation, including hyaluronic acid, procollagen III N‐terminal peptide (PIIINP), and tissue inhibitor of metalloproteinases‐1 (TIMP‐1), we were unable to calculate the ELF score in the present study. In future prospective studies, we plan to incorporate the ELF score to further evaluate liver fibrosis in patients with MASLD. Besides, we use a non‐invasive test (Fibroscan) instead of liver biopsy to evaluate the degree of liver fibrosis, which may not fully reflect the true degree of liver fibrosis in MASLD patients. Finally, due to the lack of GWAS summary‐level datasets from other regions (Asia, Africa, America, etc.) in the MR analysis, our study only explored the European population, which may lead to regional bias and lack of generalizability. Given these limitations, further high‐quality prospective cohort studies and randomized controlled trials are warranted to confirm the effect of aspirin on liver fibrosis in MASLD patients.

In conclusion, our study did not find that aspirin can significantly reduce the risk of liver fibrosis in MASLD patients based on actual observation of the American population and genetic associations by MR analysis. Therefore, long‐term use of aspirin to prevent the progression of liver fibrosis is not recommended for patients without high cardiovascular and cerebrovascular risk factors.

## Author Contributions

L.Y. and B.Z. designed the study, analyzed the data, and drafted the initial manuscript. B.Z. contributed to the revision of the manuscript. All authors read and approved the final manuscript.

## Funding

The authors have nothing to report.

## Ethics Statement

The authors have nothing to report.

## Conflicts of Interest

The authors declare no conflicts of interest.

## Supporting information


**Data S1:** fsn371384‐sup‐0001‐Supinfo.docx.

## Data Availability

These NHANES database data can be obtained here: https://wwwn.cdc.gov/nchs/nhanes/. The FinnGen study data are accessible under application at https://www.finngen.fi/en/access_results and other all data described in our study are provided within this article.

## References

[fsn371384-bib-0001] Castera, L. , M. E. Rinella , and E. A. Tsochatzis . 2025. “Noninvasive Assessment of Liver Fibrosis.” New England Journal of Medicine 393, no. 17: 1715–1729. 41160822 10.1056/NEJMra2403308

[fsn371384-bib-0002] Chalasani, N. , Z. Younossi , J. E. Lavine , et al. 2018. “The Diagnosis and Management of Nonalcoholic Fatty Liver Disease: Practice Guidance From the American Association for the Study of Liver Diseases.” Hepatology 67, no. 1: 328–357. 28714183 10.1002/hep.29367

[fsn371384-bib-0003] EASL, EASD, and EASO . 2024. “EASL‐EASD‐EASO Clinical Practice Guidelines on the Management of Metabolic Dysfunction‐Associated Steatotic Liver Disease (MASLD).” Journal of Hepatology 81, no. 3: 492–542. 38851997 10.1016/j.jhep.2024.04.031

[fsn371384-bib-0004] Emdin, C. A. , A. V. Khera , and S. Kathiresan . 2017. “Mendelian Randomization.” JAMA 318, no. 19: 1925–1926. 29164242 10.1001/jama.2017.17219

[fsn371384-bib-0005] Eslam, M. , J. G. Fan , M. L. Yu , et al. 2025. “The Asian Pacific Association for the Study of the Liver Clinical Practice Guidelines for the Diagnosis and Management of Metabolic Dysfunction‐Associated Fatty Liver Disease.” Hepatology International 19, no. 2: 261–301. 40016576 10.1007/s12072-024-10774-3

[fsn371384-bib-0006] Estes, C. , H. Razavi , R. Loomba , Z. Younossi , and A. J. Sanyal . 2018. “Modeling the Epidemic of Nonalcoholic Fatty Liver Disease Demonstrates an Exponential Increase in Burden of Disease.” Hepatology 67, no. 1: 123–133. 28802062 10.1002/hep.29466 PMC5767767

[fsn371384-bib-0007] European Association for the Study of the Liver . 2021. “EASL Clinical Practice Guidelines on Non‐Invasive Tests for Evaluation of Liver Disease Severity and Prognosis—2021 Update.” Journal of Hepatology 75, no. 3: 659–689. 34166721 10.1016/j.jhep.2021.05.025

[fsn371384-bib-0008] Haldar, D. , B. Kern , J. Hodson , et al. 2019. “Outcomes of Liver Transplantation for Non‐Alcoholic Steatohepatitis: A European Liver Transplant Registry Study.” Journal of Hepatology 71, no. 2: 313–322. 31071367 10.1016/j.jhep.2019.04.011 PMC6656693

[fsn371384-bib-0009] Jiang, Z. G. , L. Feldbrügge , E. B. Tapper , et al. 2016. “Aspirin Use Is Associated With Lower Indices of Liver Fibrosis Among Adults in the United States.” Alimentary Pharmacology & Therapeutics 43, no. 6: 734–743. 26749582 10.1111/apt.13515

[fsn371384-bib-0010] Kim, D. , W. Kim , A. C. Adejumo , et al. 2019. “Race/Ethnicity‐Based Temporal Changes in Prevalence of NAFLD‐Related Advanced Fibrosis in the United States, 2005‐2016.” Hepatology International 13, no. 2: 205–213. 30694445 10.1007/s12072-018-09926-z

[fsn371384-bib-0011] Man, S. , Y. Deng , Y. Ma , et al. 2023. “Prevalence of Liver Steatosis and Fibrosis in the General Population and Various High‐Risk Populations: A Nationwide Study With 5.7 Million Adults in China.” Gastroenterology 165: 1025–1040. 37380136 10.1053/j.gastro.2023.05.053

[fsn371384-bib-0012] Mikolasevic, I. , L. Orlic , N. Franjic , G. Hauser , D. Stimac , and S. Milic . 2016. “Transient Elastography (FibroScan()) With Controlled Attenuation Parameter in the Assessment of Liver Steatosis and Fibrosis in Patients With Nonalcoholic Fatty Liver Disease—Where Do We Stand?” World Journal of Gastroenterology 22, no. 32: 7236–7251. 27621571 10.3748/wjg.v22.i32.7236 PMC4997649

[fsn371384-bib-0013] Premkumar, M. , and A. C. Anand . 2021. “Tobacco, Cigarettes, and the Liver: The Smoking Gun.” Journal of Clinical and Experimental Hepatology 11, no. 6: 700–712. 34866849 10.1016/j.jceh.2021.07.016 PMC8617531

[fsn371384-bib-0014] Rinella, M. E. , J. V. Lazarus , V. Ratziu , et al. 2024. “A Multisociety Delphi Consensus Statement on New Fatty Liver Disease Nomenclature.” Annals of Hepatology 29, no. 1: 101133. 37364816 10.1016/j.aohep.2023.101133

[fsn371384-bib-0015] Sako, S. , Y. Takeshita , H. Takayama , et al. 2023. “Trajectories of Liver Fibrosis and Gene Expression Profiles in Nonalcoholic Fatty Liver Disease Associated With Diabetes.” Diabetes 72, no. 9: 1297–1306. 37343270 10.2337/db22-0933

[fsn371384-bib-0016] Simon, T. G. , J. Henson , S. Osganian , et al. 2019. “Daily Aspirin Use Associated With Reduced Risk for Fibrosis Progression in Patients With Nonalcoholic Fatty Liver Disease.” Clinical Gastroenterology and Hepatology: The Official Clinical Practice Journal of the American Gastroenterological Association 17, no. 13: 2776–2784.e4. 31077838 10.1016/j.cgh.2019.04.061 PMC6842070

[fsn371384-bib-0017] Sterling, R. K. , K. Patel , A. Duarte‐Rojo , et al. 2025. “AASLD Practice Guideline on Blood‐Based Noninvasive Liver Disease Assessment of Hepatic Fibrosis and Steatosis.” Hepatology 81, no. 1: 321–357. 38489523 10.1097/HEP.0000000000000845

[fsn371384-bib-0018] Suárez, M. , R. Martínez , A. M. Torres , B. Torres , and J. Mateo . 2023. “A Machine Learning Method to Identify the Risk Factors for Liver Fibrosis Progression in Nonalcoholic Steatohepatitis.” Digestive Diseases and Sciences 68, no. 9: 3801–3809. 37477764 10.1007/s10620-023-08031-y

[fsn371384-bib-0019] Thrift, A. P. , T. H. Nguyen , C. Pham , et al. 2023. “The Prevalence and Determinants of NAFLD and MAFLD and Their Severity in the VA Primary Care Setting.” Clinical Gastroenterology and Hepatology: The Official Clinical Practice Journal of the American Gastroenterological Association 21, no. 5: 1252–1260.e5. 35811043 10.1016/j.cgh.2022.05.046 PMC9825675

[fsn371384-bib-0020] Tiwari‐Heckler, S. , Z. G. Jiang , Y. Popov , and K. J. Mukamal . 2019. “Daily High‐Dose Aspirin Does Not Lower APRI in the Aspirin‐Myocardial Infarction Study.” Journal of Biomedical Research 34, no. 2: 139–142. 32305968 10.7555/JBR.33.20190041 PMC7183302

[fsn371384-bib-0021] Wu, Z. , Y. Wu , W. Zhong , et al. 2023. “The Hepatoprotective Effect of Aspirin on Carbon Tetrachloride‐Induced Hepatic Fibrosis via Inhibition of TGFβ‐1 Pathway and Pro‐Inflammatory Cytokines IL‐1β and COX‐2 in Rats.” Experimental and Therapeutic Medicine 25, no. 5: 232. 37114173 10.3892/etm.2023.11931 PMC10127207

[fsn371384-bib-0022] Xiao, G. , S. Zhu , X. Xiao , L. Yan , J. Yang , and G. Wu . 2017. “Comparison of Laboratory Tests, Ultrasound, or Magnetic Resonance Elastography to Detect Fibrosis in Patients With Nonalcoholic Fatty Liver Disease: A Meta‐Analysis.” Hepatology 66, no. 5: 1486–1501. 28586172 10.1002/hep.29302

[fsn371384-bib-0023] Younossi, Z. , Q. M. Anstee , M. Marietti , et al. 2018. “Global Burden of NAFLD and NASH: Trends, Predictions, Risk Factors and Prevention.” Nature Reviews Gastroenterology & Hepatology 15, no. 1: 11–20. 28930295 10.1038/nrgastro.2017.109

[fsn371384-bib-0024] Younossi, Z. , M. Stepanova , J. P. Ong , et al. 2019. “Nonalcoholic Steatohepatitis Is the Fastest Growing Cause of Hepatocellular Carcinoma in Liver Transplant Candidates.” Clinical Gastroenterology and Hepatology: The Official Clinical Practice Journal of the American Gastroenterological Association 17, no. 4: 748–755.e3. 29908364 10.1016/j.cgh.2018.05.057

[fsn371384-bib-0025] Younossi, Z. M. , A. B. Koenig , D. Abdelatif , Y. Fazel , L. Henry , and M. Wymer . 2016. “Global Epidemiology of Nonalcoholic Fatty Liver Disease‐Meta‐Analytic Assessment of Prevalence, Incidence, and Outcomes.” Hepatology 64, no. 1: 73–84. 26707365 10.1002/hep.28431

