# Supplementary material for: Aspirin Use Does Not Significantly Reduce the Risk of Liver Fibrosis in Patients With Metabolic Dysfunction‐Associated Steatotic Liver Disease
Source: Food Sci Nutr. 2025 Dec 26;14(1):e71384. doi: 10.1002/fsn3.71384 (PMC12741777; doi:10.1002/fsn3.71384)
Supplement: Supplementary file 1 — Data S1: fsn371384‐sup‐0001‐Supinfo.docx. [file FSN3-14-e71384-s001.docx]

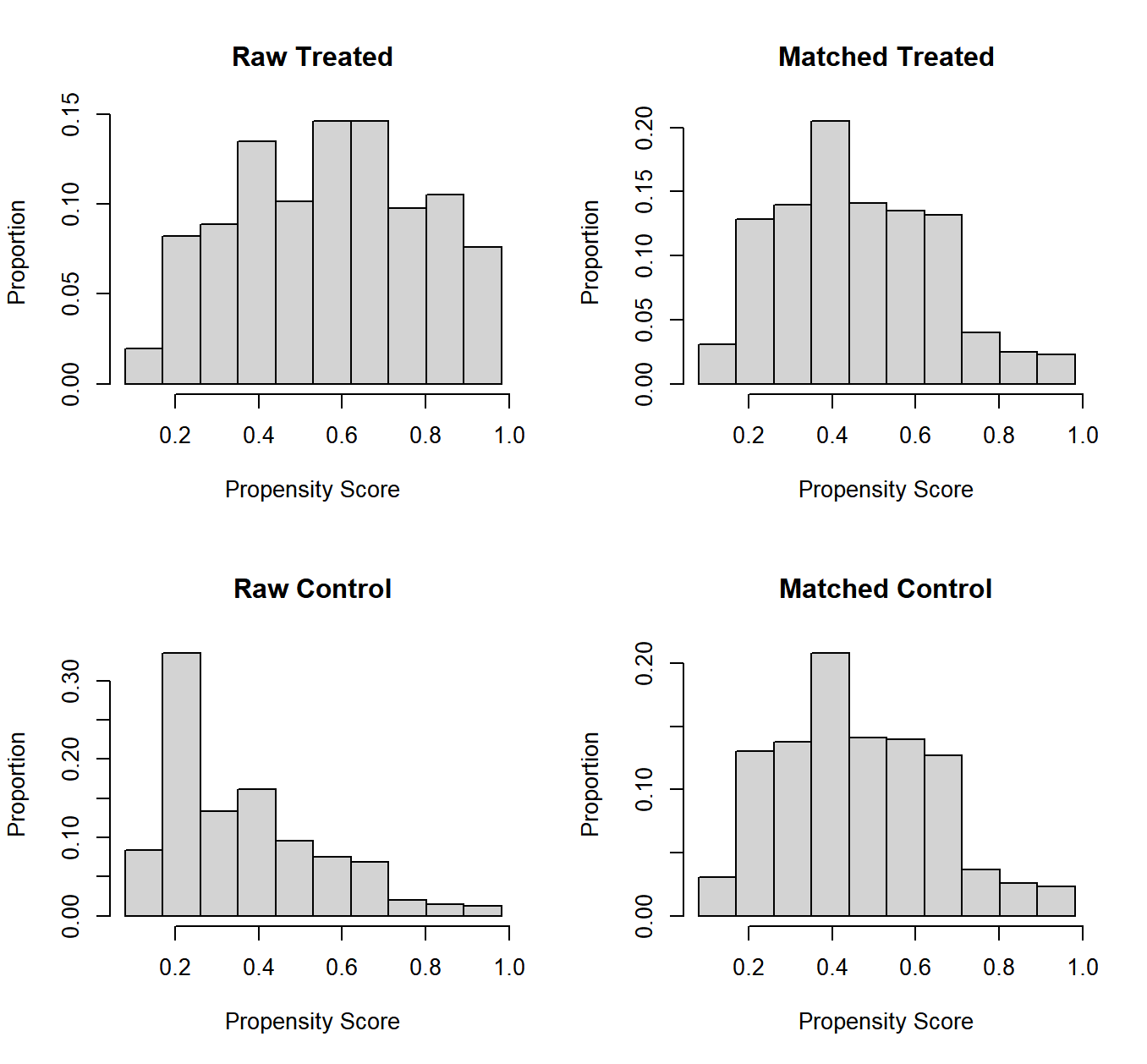


**Supplementary Figure 1**: The histograms of propensity scores in MASLD patients


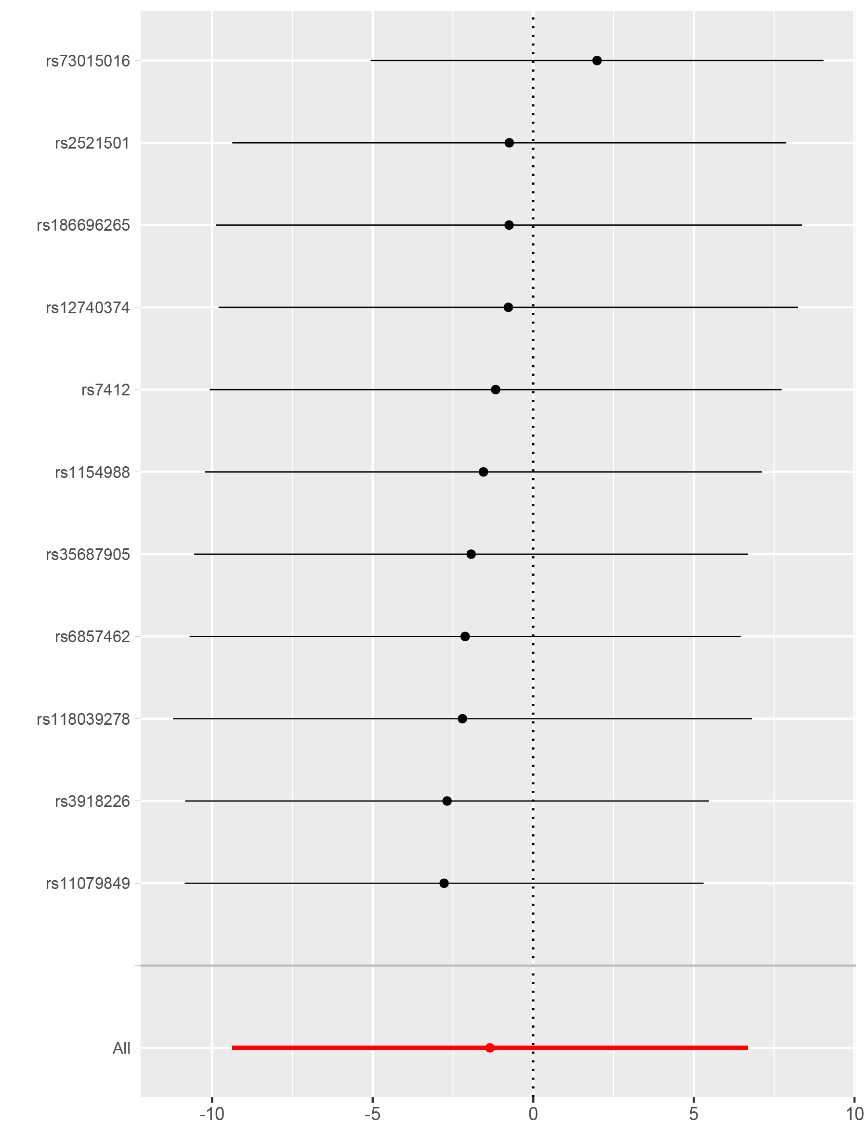


**Supplementary Figure 2**: MR leave-one-out sensitivity analysis for aspirin on fibrosis and cirrhosis of the liver

**Supplementary Table 1:** SNPs of aspirin in the Mendelian randomization analysis

| **SNP** | **Chr** | **Pos** | **Beta** | **SE** | **P** | **Sample size** | **EA** | **OA** | **EAF** | **R2** | **F** |
| --- | --- | --- | --- | --- | --- | --- | --- | --- | --- | --- | --- |
| rs12740374 | 1 | 109817590 | -0.006 | 0.001 | 2.20E-14 | 462933 | T | G | 0.221 | 1.26E-04 | 58.348 |
| rs1154988 | 3 | 135925191 | 0.005 | 0.001 | 4.00E-08 | 462933 | A | T | 0.773 | 6.51E-05 | 30.137 |
| rs6857462 | 4 | 52695704 | 0.004 | 0.001 | 3.40E-08 | 462933 | G | T | 0.294 | 6.58E-05 | 30.457 |
| rs118039278 | 6 | 160985526 | 0.013 | 0.001 | 6.10E-24 | 462933 | A | G | 0.079 | 2.20E-04 | 101.805 |
| rs186696265 | 6 | 161111700 | 0.022 | 0.003 | 2.10E-14 | 462933 | T | C | 0.015 | 1.26E-04 | 58.432 |
| rs3918226 | 7 | 150690176 | 0.008 | 0.001 | 3.90E-09 | 462933 | T | C | 0.081 | 7.49E-05 | 34.686 |
| rs35687905 | 8 | 74826565 | 0.006 | 0.001 | 3.60E-08 | 462933 | C | T | 0.113 | 6.56E-05 | 30.365 |
| rs1831733 | 9 | 22076071 | 0.007 | 0.001 | 4.70E-22 | 462933 | C | T | 0.478 | 2.01E-04 | 93.209 |
| rs7310615 | 12 | 111865049 | -0.004 | 0.001 | 5.40E-10 | 462933 | G | C | 0.518 | 8.32E-05 | 38.512 |
| rs2521501 | 15 | 91437388 | 0.004 | 0.001 | 2.10E-08 | 462933 | T | A | 0.322 | 6.77E-05 | 31.357 |
| rs11079849 | 17 | 47090785 | -0.004 | 0.001 | 8.10E-09 | 462933 | T | C | 0.329 | 7.18E-05 | 33.260 |
| rs7412 | 19 | 45412079 | -0.010 | 0.001 | 8.00E-15 | 462933 | T | C | 0.080 | 1.30E-04 | 60.332 |
| rs73015016 | 19 | 11191300 | -0.007 | 0.001 | 5.20E-12 | 462933 | A | G | 0.119 | 1.03E-04 | 47.597 |
| rs8126001 | 20 | 62711459 | -0.004 | 0.001 | 2.40E-08 | 462933 | T | C | 0.490 | 6.73E-05 | 31.141 |

**Supplementary Table 2:** Association of genome-wide SNPs for aspirin with fibrosis and cirrhosis of the liver

| **SNP** | **Chr** | **Pos** | **Beta** | **SE** | **P** | **EAF** | **EA** | **OA** |
| --- | --- | --- | --- | --- | --- | --- | --- | --- |
| rs12740374 | 1 | 109817590 | 0.034 | 0.061 | 0.584 | 0.215 | T | G |
| rs1154988 | 3 | 135925191 | 0.012 | 0.071 | 0.871 | 0.852 | A | T |
| rs6857462 | 4 | 52695704 | 0.038 | 0.055 | 0.494 | 0.288 | G | T |
| rs118039278 | 6 | 160985526 | 0.051 | 0.120 | 0.670 | 0.046 | A | G |
| rs186696265 | 6 | 161111700 | -0.107 | 0.199 | 0.591 | 0.017 | T | C |
| rs3918226 | 7 | 150690176 | 0.124 | 0.099 | 0.209 | 0.070 | T | C |
| rs35687905 | 8 | 74826565 | 0.044 | 0.083 | 0.593 | 0.101 | C | T |
| rs7310615 | 12 | 111865049 | -0.195 | 0.051 | 0.000 | 0.586 | G | C |
| rs2521501 | 15 | 91437388 | -0.042 | 0.057 | 0.455 | 0.267 | T | A |
| rs11079849 | 17 | 47090785 | -0.074 | 0.055 | 0.181 | 0.299 | T | C |
| rs7412 | 19 | 45412079 | 0.030 | 0.111 | 0.784 | 0.054 | T | C |
| rs73015016 | 19 | 11191300 | 0.249 | 0.083 | 0.003 | 0.103 | A | G |
